# The Horse in Motion

**Published:** 1898-05

**Authors:** 


### REVIEW.

THE HORSE IN MOTION. Drawings by WILLIAM HAHN. By DR. J. D. B. STILLMAN. Preface by the late LELAND STANFORD. A book of nine beautifully colored plates, ninety to one hundred pages of heliotype cuts, one hundred pages of text. Printed upon a heavy, rich, plate paper, nine by twelve inches; bound in cloth, with gilt top. Published originally as a subscription book at ten dollars. The remaining copies (the plates having been destroyed) are now selling at five dollars. Ticknor & Co. 211 Tremont St., Boston, Mass.

This work and the field it represents has been, since the issue of this excellent work, one of the most interesting, instructive, and important lines of investigation, and has marked an era of advance of great value. Instantaneous photography has revealed the gaits of horses and animals and made possible many attainments in the study of locomotion that have added an untold measure of results in dealing with the advances made and attained in breeding centres. The late Mr. Leland Stanford found this a field of the greatest promise in reaching ends in breeding, and this work, published largely under his fostering direction, has done much to extend investigation along these lines. It is a magnificent book, of the greatest interest to veterinarians, and should be one of those books owned by every member of the prefession, for consultation and interesting reading at odd moments of leisure.

